# The habitat-modifying red alga *Ramicrusta* on Pacific reefs: A new generic record for the Tropical Northwestern Pacific and the description of four new species from Guam

**DOI:** 10.1371/journal.pone.0259336

**Published:** 2021-11-15

**Authors:** Matthew S. Mills, Tom Schils

**Affiliations:** 1 Marine Laboratory, University of Guam, Mangilao, Guam; 2 School of Science, Technology, and Engineering, University of the Sunshine Coast, Sippy Downs, Queensland, Australia; Federal University of Bahia: Universidade Federal da Bahia, BRAZIL

## Abstract

The genus *Ramicrusta* (order Peyssonneliales) is a new record for Micronesia, with range expansions of *Ramicrusta fujiiana* and *R*. *lateralis* to Guam. In addition, four species (*Ramicrusta adjoulanensis*, *R*. *asanitensis*, *R*. *labtasiensis*, and *R*. *taogamensis*) are newly described from Guam using molecular and anatomical characters. *Ramicrusta lateralis* specimens from Guam share most anatomical features with the holotype description from Vanuatu, but the plants from Guam are more tightly adherent, rigid, and robust than those of Vanuatu. *Ramicrusta adjoulanensis* possesses a well-developed epithallus with frequent cell fusions, secondary pit connections, and lacking hair bases or trichocytes, similar to *Ramicrusta bonairensis*. *Ramicrusta adjoulanensis* differs from other *Ramicrusta* species in having occasionally free margins and being attached by frequently produced, relatively long rhizoids (75–100 μm long). *Ramicrusta asanitensis* shares features with many other species, but the thickness of the crust (upwards of 2 mm thick), heavy calcification in the epithallus, and the extent of secondary, tertiary, and quaternary growth, differentiate it from other *Ramicrusta* species. *Ramicrusta labtasiensis* shares features with its close relative *Ramicrusta lateralis* but possesses frequent, robust, and relatively long rhizoids (75–95 μm long) throughout its entire undersurface. *Ramicrusta taogamensis* resembles its close relative *Ramicrusta appressa* but is primarily distinguished by its generally well-developed epithallus with occasional secondary pit connections and cell fusions. The six species reported here make Guam equal to Vanuatu in currently having the highest known species richness of *Ramicrusta* in the world.

## Introduction

Among crustose calcifying red algae (CCRA), calcifying and encrusting members of the Peyssonneliales [1] have historically been overlooked in favor of the more frequently studied members of the Corallinophycidae. However, advances in molecular techniques have greatly benefitted studies of CCRA diversity and systematics [2, 3]. Recently, however, the Peyssonneliales have received more attention and recognition in part due to their ecological [4–10] significance. Members of the Peyssonneliales are distributed circumglobally, occurring from shallow intertidal waters to depths greater than 250 m [1, 6, 9]. Recent studies have reported some members of the Peyssonneliales comprising significant portions of benthic reef habitats [4, 5, 7] and inhibiting coral growth [10], while another documented their potential to be resilient in the face of future ocean acidification [8].

There are currently 13 recognized genera in the Peyssonneliales [11]. One of these genera, *Ramicrusta* [12], was initially distinguished from other Peyssonneliales crusts by possessing secondary pit connections. More recently, *Ramicrusta* has been further distinguished from *Peyssonnelia* using a suite of additional vegetative characters such as a combination of secondary pit connections, cell fusions, and unicellular rhizoids [13] in support of its generic status based on phylogenetic inference. One such vegetative feature, initially referred to as ‘heterocysts’ [12], are present near the dorsal surface in the majority of *Ramicrusta* species. Pueschel and Saunders [14] hypothesized that these enlarged cells embedded in the perithallus were the persistent bases of shed hairs. When present, these hair bases are often, but not always, much larger than neighboring filaments, bullet-shaped, and terminate filaments of two to seven cells [12–15]. Following that interpretation, they have conventionally been referred to as ‘hairs’ or ‘hair bases’ when describing new *Ramicrusta* species [13, 15]. However, analogous structures in the Corallinophycidae are known as trichocytes [16, 17] and, following the convention introduced by Ballantine et al. [15], we will refer to these structures as ‘hair bases or trichocytes’, or simply ‘hair bases’, herein.

*Ramicrusta nanhaiensis* D.R.Zhang & J.H.Zhou, the type species of *Ramicrusta*, was described from the Paracel Islands in the South China Sea [12]. Since then, 14 additional species have been described or transferred to *Ramicrusta* [11]. Six of these species were described from Vanuatu and Australia in the southern Pacific [13]. Additionally, *Peyssonnelia calcea* Heydrich, a species described from Papua New Guinea with a Pacific-wide distribution, was transferred to *Ramicrusta* [13]. The remaining seven species were described from the Caribbean Sea and the Hawaiian Islands [11]. The first species of *Ramicrusta* known from the western Atlantic, *Ramicrusta textilis* Pueschel & G.W.Saunders, was described in 2009 from nearshore reefs in Jamaica [14] and was later reported for Puerto Rico [18], Vanuatu [13], and Taiwan [19]. In 2016, *Ramicrusta monensis* Ballantine, Ruiz, Lozada-Troche & Norris was described from Puerto Rico, and *Ramicrusta bonairensis* Ballantine, Ruiz, Lozada-Troche & Norris was described from Bonaire and has been reported for Puerto Rico [15]. In 2018, *Ramicrusta melanoidea* K.R.Dixon was described from northwestern Australia and Vanuatu [20] as a *Ramicrusta* species based on its morphological features, but molecular data suggests that the species might be better placed in a different genus [21]. *Ramicrusta fujiiana* E.M.S.Pestana, G.N.Santos, Cassano & J.M.C. Nunes and *Ramicrusta paradoxa* E.M.S.Pestana, G.N.Santos, Cassano & J.M.C.Nunes were recently described from southeastern Brazil [22]. Most recently, *Ramicrusta hawaiiensis* A.R.Sherwood and *Ramicrusta lehuensis* A.R.Sherwood, were described from Lehua Island in Hawaii [23]. Finally, a yet undescribed species of *Ramicrusta* was reported from Tunisia, representing the first report of the genus in the Mediterranean [24].

In the Caribbean, *R*. *textilis* is known to rapidly overgrow living coral, which can result in a significant loss of living coral and associated organisms [18]. However, the composition of macro-invertebrate and fish communities associated with *Ramicrusta*-dominated reefs in Puerto Rico are reportedly similar to those of scleractinian-dominated patch reefs [15, 21]. *R*. *textilis* has also been documented to overgrow both dead and living coral colonies of shallow reefs in Dongsha Atoll, South China Sea. Here, *R*. *textilis* forms a species community with other crustose algae (*e*.*g*., Peyssonneliales and *Lobophora* spp.) that can cover up to 29% of the benthic substrate [19]. *R*. *bonairensis* has also been observed overgrowing corals and sponges on disturbed Caribbean reefs [25, 26].

Below, we describe four new species of *Ramicrusta* from Guam based on comparative genetic and morphological analyses. *R*. *fujiiana* and *R*. *lateralis* are also reported as new species records for Guam. These are the first records of the genus *Ramicrusta* for the Tropical Northwestern Pacific marine province [27].

## Materials and methods

### Collection and morphological analysis

**Fig 1:**
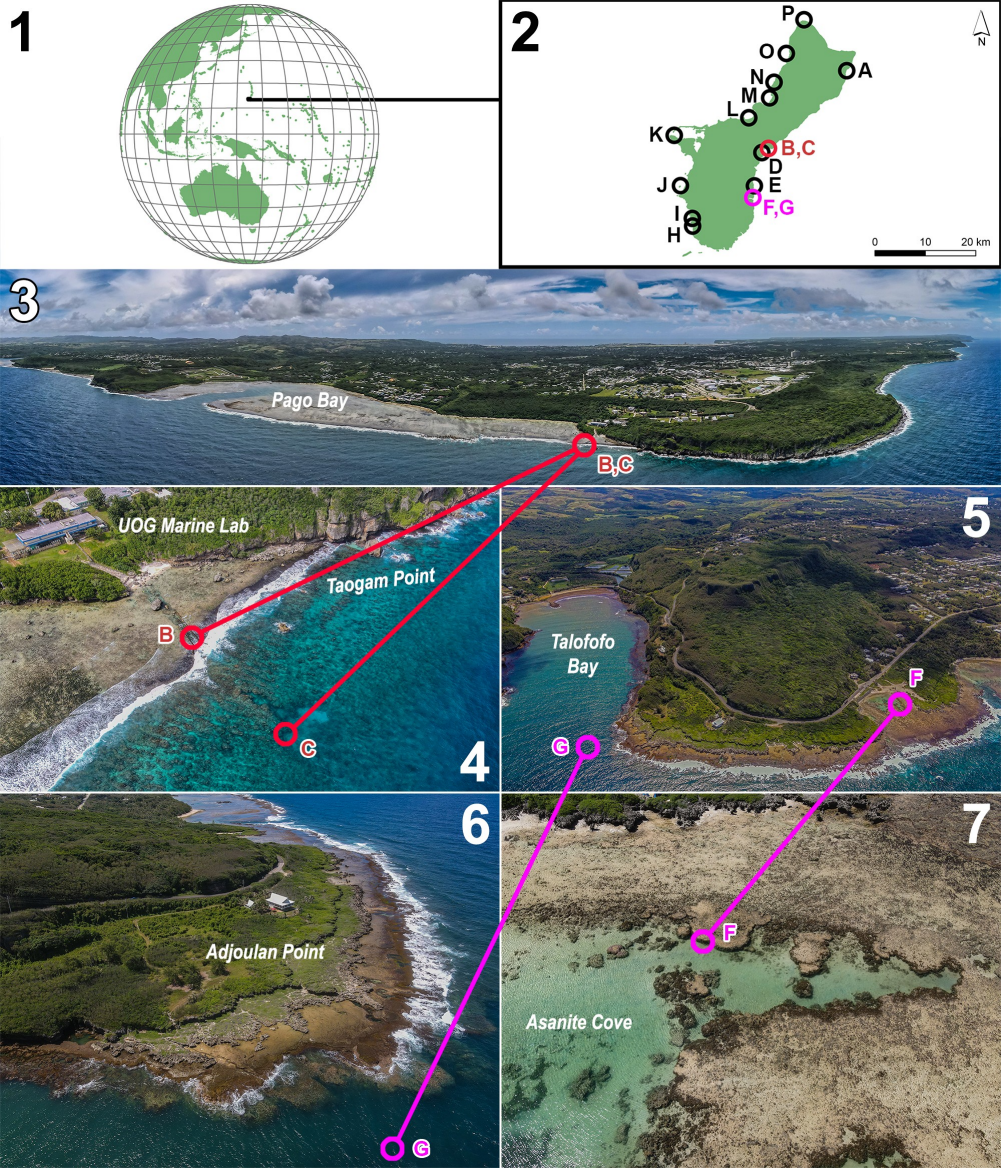
Maps indicating the study area and sample collection locations. (1) Pacific-centered map indicating the location of the island of Guam. (2) Map of Guam identifying the sites from which *Ramicrusta* specimens were collected. Type localities of the four species being described are in red or pink. Scale bar = 20 km. (3–4) Aerial photographs depicting the type localities of *R*. *labtasiensis* (Point # B) and *R*. *taogamensis* (Point # C). (5–7) Aerial photographs showing the type localities of *R*. *adjoulanensis* (Point # G) and *R*. *asanitensis* (Point # F). Maps were created using the ArcGIS computer software (Esri, Redlands, CA), and aerial photographs were captured on-site using a drone.

Samples were collected by reef wading, snorkeling, and diving at various sites around Guam (Fig 1). Collection permits were obtained from the Guam Department of Agriculture’s Division of Aquatic and Wildlife Resources (DAWR). Specimens were photographed *in situ*, collected, and photographed again before being transferred to holding tanks with running seawater until DNA extraction. Portions of samples were preserved in formalin, silica gel, and air-dried as herbarium specimens. Specimens were deposited at the University of Guam Herbarium (GUAM). However, the *Ramicrusta fujiiana* specimen that was collected, photographed, and sequenced was lost from the holding tank before it could be preserved. As such, the report of *R*. *fujiiana* for Guam is based on the DNA sequence data obtained from the specimen before it was lost. For anatomical observations, material was hand-sectioned using a razor blade and embedded on 12.7 mm pin mounts using colloidal graphite with isopropanol base (Energy Beam Sciences). The sections were sputter coated using an Emitech SC7620 Sputter Coater (Quorum Technologies Ltd., Laughton, East Sussex, United Kingdom). Anatomical observations were made and imaged using a Phenom G2 Pro desktop scanning electron microscope (Phenom-World B.V., Eindhoven, The Netherlands).

### Molecular analysis

For molecular analyses, total genomic DNA was extracted using the QIAGEN DNeasy Blood & Tissue Kit (Qiagen Inc., Valencia, CA) or the GenCatch Blood & Tissue Genomic Mini Prep Kit (Epoch Life Science Inc., Missouri City, TX) following the manufacturers’ bench protocol. The mitochondrial COI-5P was polymerase chain reaction (PCR) amplified using a newly designed forward primer TS_COI_F01_10 (5’- TCGARTCYCGTCTCTCTCG -3’) and the reverse primer GWSRx [28] following the amplification profile 95°C for 3 minutes; 35 cycles of 94°C for 40 seconds, annealing at 48°C for 40 seconds, extension at 72°C for 100 seconds; a final extension at 72°C for 10 minutes. Chloroplast *psb*A was amplified using the primers developed by Yoon et al. [29] following the amplification profile 95°C for 3 minutes; 35 cycles of 94°C for 40 seconds, annealing at 50°C for 40 seconds, extension at 72°C for 100 seconds; a final extension at 72°C for 10 minutes. Plastid *rbc*L was amplified using the forward primer F57 [30] and the reverse primer rbcLrevNEW [31] following the amplification profile reported by Saunders & Moore [31]. PCR products were sent to Macrogen Inc. (Seoul, Republic of Korea) for purification and DNA sequencing.

Alignments for each of the gene regions were created using the MUSCLE plugin [32] in Geneious Pro 11.0.5 [33]. The COI-5P, *rbc*L, and *psb*A alignments were all analyzed independently prior to a combined analysis of all three genes. An alignment of fifty-one homologous COI-5P sequences was used to establish the phylogenetic relationship of *Ramicrusta* species from Guam and all but two currently described species of the genus. Lack of available sequence data excluded *Ramicrusta calcea* from phylogenetic analyses, while *Ramicrusta melanoidea* was excluded because of its high average COI-5P sequence divergence with other *Ramicrusta* species. Individual analyses of *rbc*L and *psb*A were limited by a lack of sequences available for comparison. A combined analysis of all three genes was used to establish phylogenetic relationships within the genus *Ramicrusta*. 51 new DNA sequences were generated for *Ramicrusta* specimens from Guam, of which 24 COI (MW960726—MW960749), 8 *rbc*L (MW960750—MW960757), and 19 *psb*A sequences (MW960758—MW960776; S1 Table). For all alignments, the general time reversal + invariable sites + gamma distribution (GTR+I+G) evolutionary model was selected as the optimal model using jModeltest 2.1.3 [34]. The concatenated alignment used in the combined analysis was partitioned by gene, and GTR+I+G was selected as the optimal model for each partition in the alignment. Phylogenetic analyses were performed for all alignments using maximum likelihood (ML) methods in RAxML [35]. The proportion of invariable sites and gamma shape parameters were estimated from the data, and sequence divergence was calculated using the MEGA version X computer software [36]. Sequence divergence was calculated using a neighbor-joining algorithm under a Kimura 2-parameter substitution model, which has been most often used when describing or reporting new *Ramicrusta* species [13, 19, 23]. Nonparametric bootstrapping (1000 replicates) was used to estimate node support. Bayesian inference was completed for each alignment using the MrBayes 3.1.2 [37] plugin in Geneious Pro 11.0.5 [32]. Each alignment was run for 1,000,000 generations with trees sampled every 100 generations, and the first 3,000 trees were discarded as burn-in (average standard deviation of split frequencies < 0.01). All COI-5P, *rbc*L, and *psb*A sequences obtained were deposited in GenBank (S1 Table), and once released, all COI-5P and *rbc*L sequences will also be available in the Barcode of Life Database (BOLD) [38].

### Nomenclature

The electronic version of this article in Portable Document Format (PDF) in a work with an ISSN or ISBN will represent a published work according to the International Code of Nomenclature for algae, fungi, and plants, and hence the new names contained in the electronic publication of a PLoS article are effectively published under that Code from the electronic edition alone, so there is no longer any need to provide printed copies.

In addition, new names contained in this work have been submitted to World Register of Marine Species (WoRMS), from where they will be made available to the Global Names Index. The WoRMS LSIDs can be resolved and the associated information viewed through any standard web browser by appending the LSID contained in this publication to the prefix http://marinespecies.org/. The online version of this work is archived and available from the following digital repositories: PubMed Central, LOCKSS.

## Results

### Molecular and phylogenetic results

**Fig 2:**
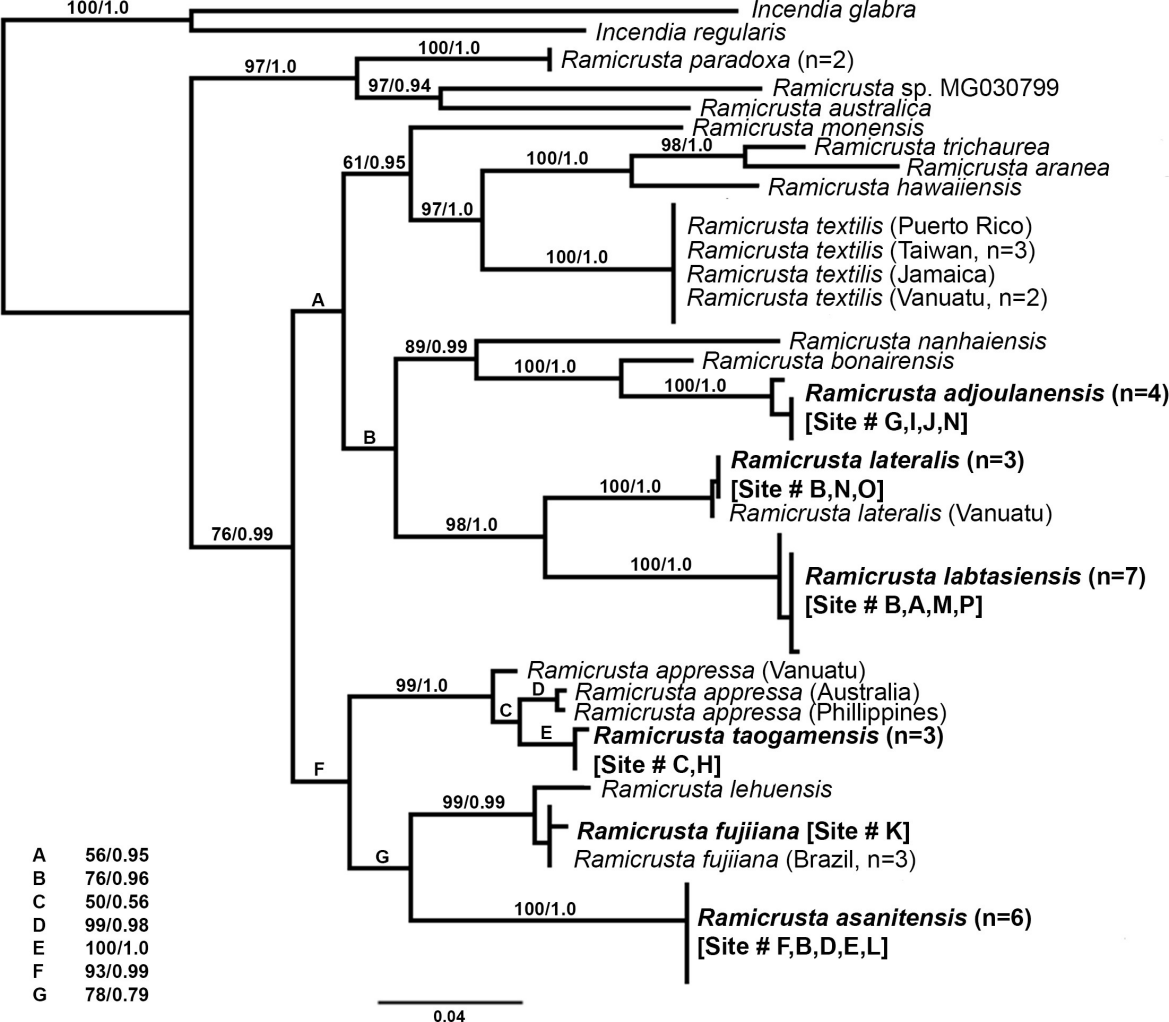
Bayesian inference phylogenetic tree of 51 COI-5P sequences, representing 18 *Ramicrusta* species and two *Incendia* species as outgroups. Bootstrap support and Bayesian posterior probability values are printed at each node (bootstrap support/posterior probability). Newly described species are in bold type.

**Fig 3:**
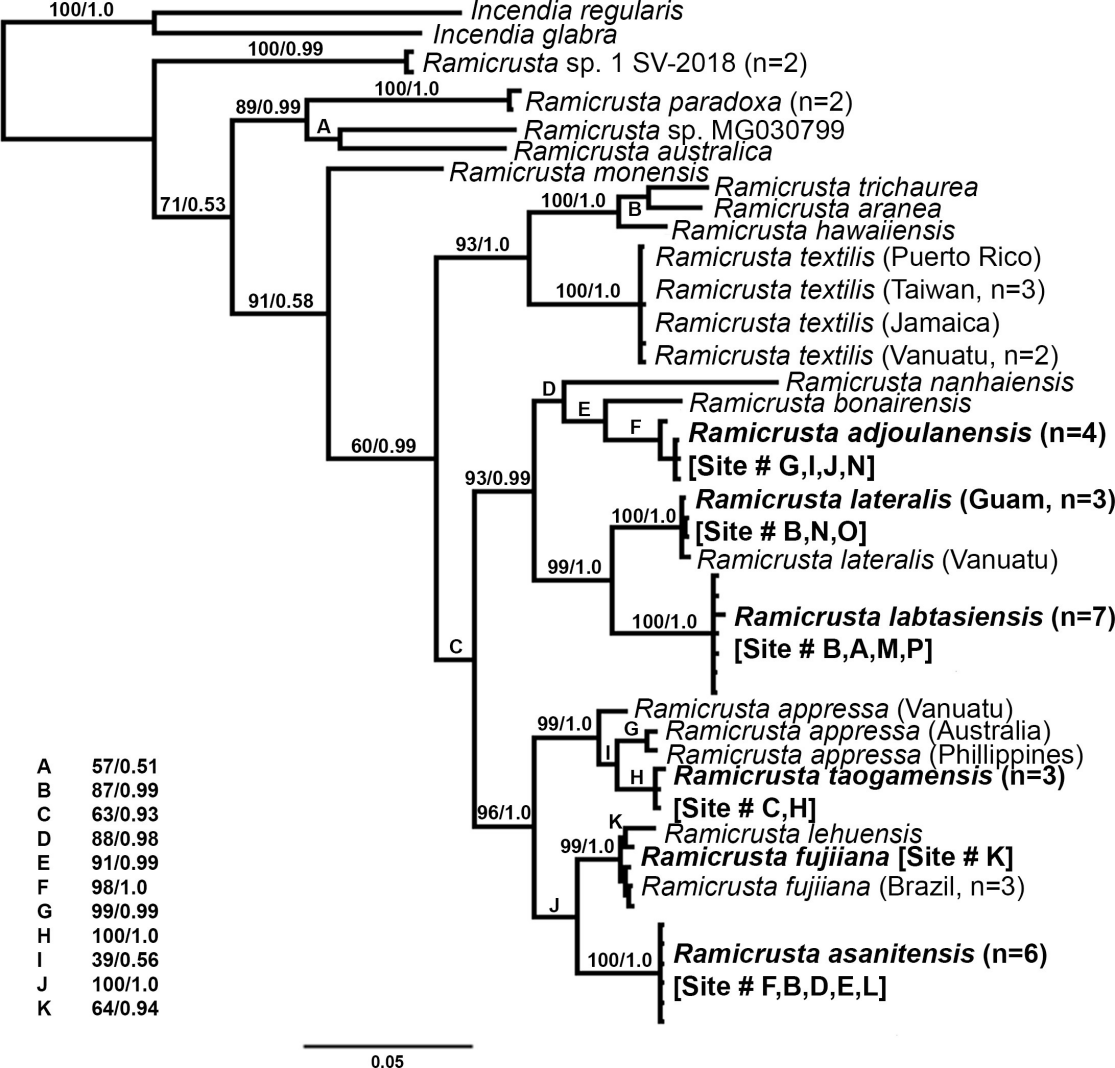
Bayesian inference phylogenetic tree of a partitioned alignment of COI-5P, *psb*A, and *rbc*L sequences, representing 19 *Ramicrusta* species and two *Incendia* species as outgroups. Bootstrap support and Bayesian posterior probability values are printed at each node (bootstrap support/posterior probability). Newly described species are in bold type.

Phylogenetic analyses of the official barcode marker for red algae, COI-5P, supported the recognition of four new *Ramicrusta* species from Guam (Fig 2). *Rbc*L and *psb*A phylogenies also support the recognition of four new *Ramicrusta* species, but the lack of sequences for previously described species does not allow for a comprehensive evaluation of phylogenetic relationships (S1 and S2 Figs). Analysis of the partitioned COI-5P, *rbc*L, and *psb*A alignment was congruent with the most taxon-complete COI-5P analysis (Fig 3).

### Taxonomic analyses

#### *Ramicrusta fujiiana* E.M.S.Pestana, G.N.Santos, Cassano et J.M.C.Nunes

**Fig 4:**
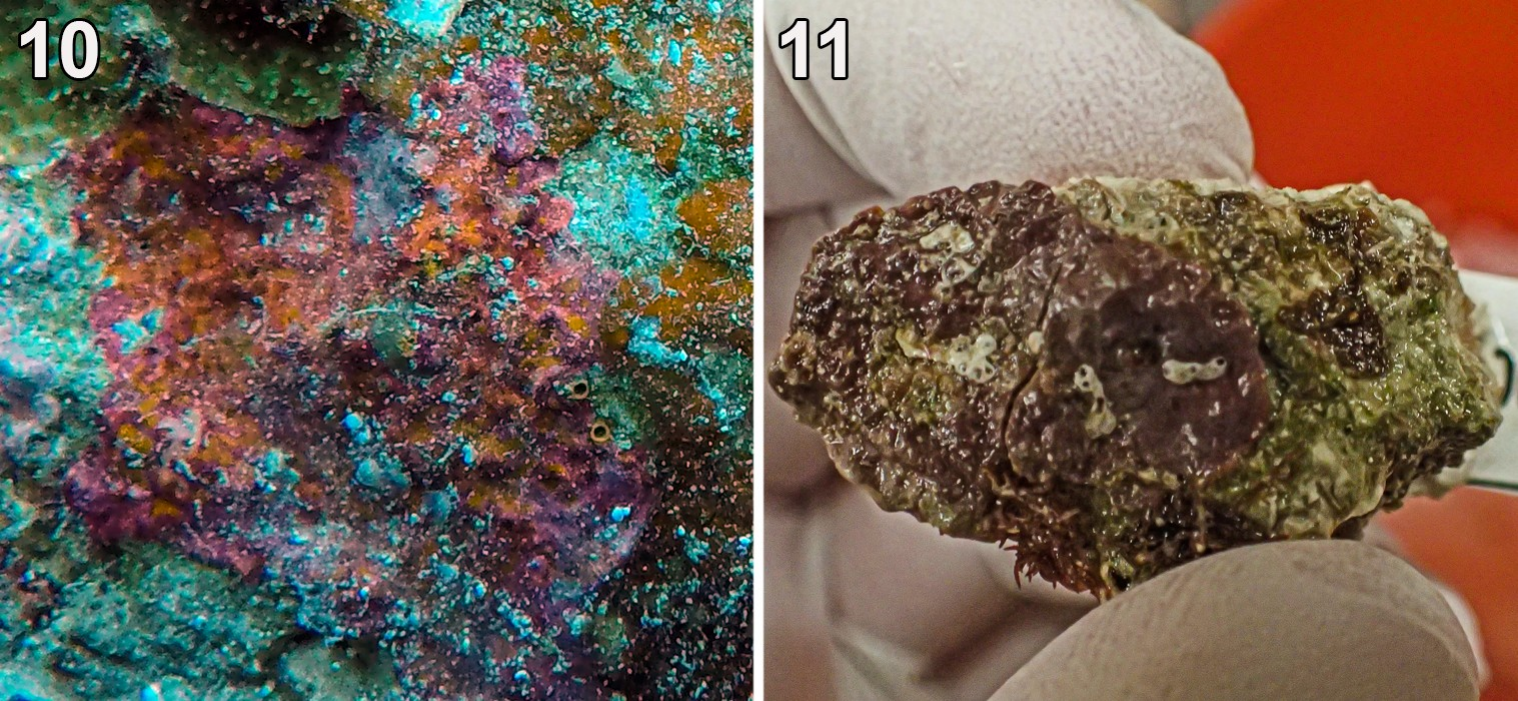
*Ramicrusta fujiiana* (GH0015078). (10) *In-situ* image of the specimen prior to collection. (11) Habit of the specimen.

(in Pestana et al., 2020: 39–55, Fig 2A–2E) Fig 4.

**Type locality:** Maraú, Algodões (14°04’15,06”S–38°57’32,05”W), Bahia, Brazil [22].

**Specimen examined:** *GH0015078*, Orote Point, Apra Harbor, Guam, Mariana Islands, western Pacific Ocean, 8.0 m depth, coll. T. Schils & M. Mills, 22.vi.2017.

Thallus was orangish-purple, completely calcified, closely appressed, and was tightly adherent to the substratum (Fig 4). The habit of the Guam specimen differed from the reddish-orange specimens from Brazil, but *R*. *fujiiana* specimens from Guam and Brazil shared their strong adherence to the substratum [22]. The Guam specimen was unfortunately lost from the holding tank before further anatomical observations could be completed, so the report of *R*. *fujiiana* for Guam is based on DNA sequence data. The COI-5P barcode sequences of Guam sample was nearly identical, with a maximum 0.36% and average 0.08% intraspecific sequence divergence to the *R*. *fujiiana* specimens from Brazil. Phylogenetic analyses supported the report of *R*. *fujiiana* for Guam based on DNA sequence data (Figs 2 and 3). The difference in environment between the sampling locations in Guam and Brazil could explain the differences in habit between these genetically equivalent plants.

#### *Ramicrusta lateralis* K.R.Dixon

(in Dixon & Saunders, 2013: 82–108, Figs 57–62)

Fig 5.

**Fig 5:**
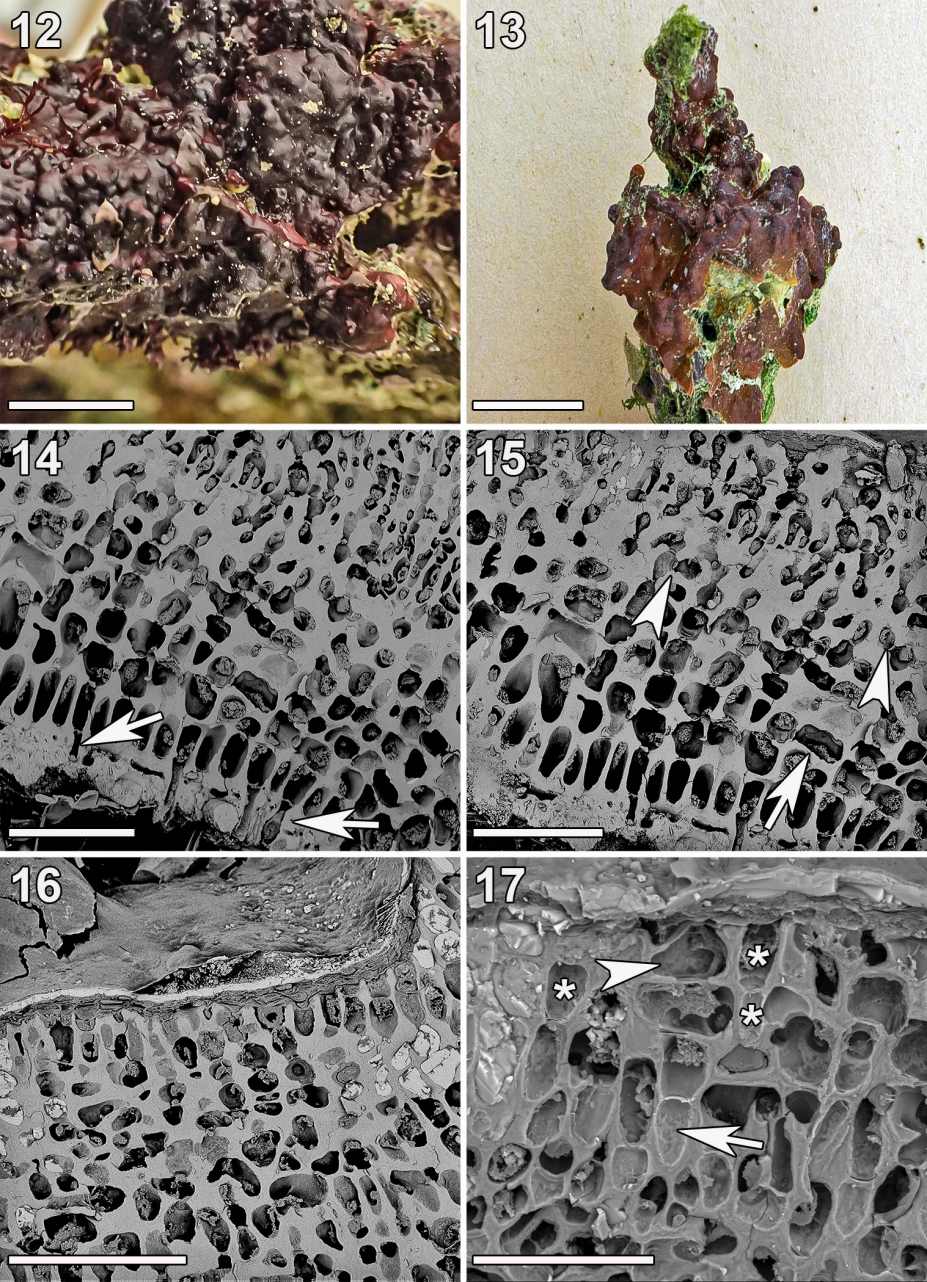
***Ramicrusta lateralis* (Fig 5: GH0015072; Fig 5: GH0015212).** (12) Habit of a specimen. Scale bar = 1 cm. (13) Crustose thallus occasionally free at the margins. Scale bar = 2 cm. (14) Frequent unicellular rhizoids (arrows) penetrating the hypobasal cuticle. Scale bar = 100 μm. (15) Radial section through crust showing secondary pit connections (arrowheads) and cell fusions (arrow) in the lower perithallus. Scale bar = 100 μm. (16) Radial section showing thin epithallus. Scale bar = 100 μm. (17) Radial section showing a large bullet-shaped hair base or trichocyte (arrowhead) terminating a filament of four cells (arrow) that are significantly larger than other surrounding apical cells (asterisks). Scale bar = 50 μm.

**Type locality:** Imenaka Reef (19.46389°S; 169.223056°E), Loanpekel, Whitegrass, Tanna, Vanuatu, South Pacific Ocean [13].

**Specimens examined:** *GH0015212*, Tanguisson reef flat, Guam, Mariana Islands, western Pacific Ocean, 0.5–1.0 m depth, coll. T. Schils, M. Deinhart & K. Borja, 25.v.2018; *GH0015072*, reef flat outside of the University of Guam Marine Laboratory, Pago Bay, Guam, Mariana Islands, western Pacific Ocean, 0.5–1.0 m depth, coll. T. Schils & M. Mills, 15.iv.2017.

Thalli were brown to reddish brown and heavily calcified (Fig 5). Crusts were 225–550 μm thick and closely appressed. Typically, crusts were tightly adherent but loosely attached around some of the margins (Fig 5). Hypothallial filaments were parallel and composed of dorsally inflated oval cells that gave rise to assurgent perithallial filaments at broad angles. Plants were attached by squat, robust, thick-walled unicellular rhizoids (c. 50 μm long, 12–16 μm wide), which originated from the ventral portion of hypothallial cells and penetrated the thick (15–25 μm) hypobasal cuticle (Fig 5). Perithallial filaments were simple or occasionally irregularly branched. Portions of secondary growth as well as overgrowth were present. Secondary growth could be recognized as alternating stacked layers of epithalli and lower perithalli (absence of hypothallial layers), while overgrowth appeared as two fully formed thalli stacked atop one another. Cells of the lower perithallus were thick walled, heavily calcified, and were frequently connected to adjacent cells via fusion or secondary pit connections (Fig 5). The epithallus was thin, lacked secondary pit connections and cell fusions, and was composed of three to four tiers of small rectilinear cells (Fig 5). Hair bases or trichocytes embedded in the upper perithallus were large (c. 20 μm long and c. 23 μm wide), bullet-shaped, and terminated filaments of three or four cells (Fig 5). Reproduction was not observed.

The COI-5P barcode sequences of the four Guam samples were nearly identical (average 0.08% intraspecific sequence divergence) to that of the holotype of *R*. *lateralis*. They also shared anatomical features such as the structure of the epithallus, perithallial filaments being borne from the hypothallus at broad angles and having portions of secondary growth. There were, however, differences in their gross morphologies: the Guam specimens were tightly adherent to the substrate throughout, while only free around some of the margins. Crusts of the Guam plants were typically thinner, but they were rigid and robust, as opposed to being brittle in Vanuatu [13]. The difference in environment between the sampling locations in Guam and Vanuatu could explain the morphological differences between these genetically equivalent plants.

#### *Ramicrusta adjoulanensis* M.Mills et Schils sp. nov.

[urn:lsid:marinespecies.org:taxname:XXXXXXX]

Fig 6.

**Fig 6:**
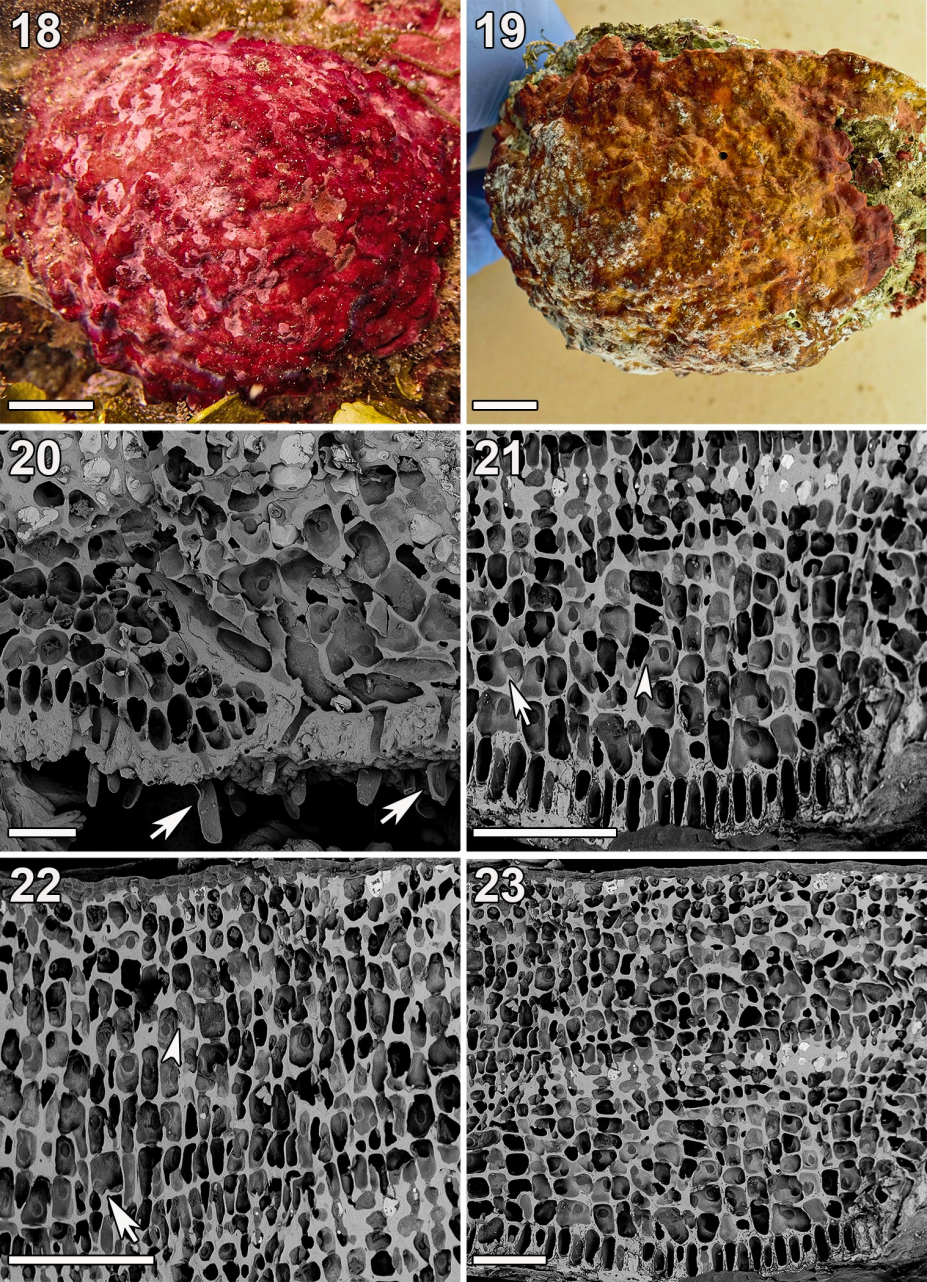
*Ramicrusta adjoulanensis* (GH0015334). (18) *In-situ* image of the holotype specimen. Scale bar = 2 cm. (19) Habit of the holotype specimen. Scale bar = 2 cm. (20) Radial section of a free margin showing frequently produced unicellular rhizoids (arrows) penetrating the thick hypobasal cuticle. Scale bar = 100 μm. (21) Section showing secondary pit connections (arrowhead) and cell fusions (arrow) in the lower perithallus, as well as the rapid decrease in cell size around the mid-perithallus. Scale bar = 100 μm. (22) Well-developed epithallus with frequently branching filaments whose cells are commonly pit connected (arrowhead) or fused (arrow) with those of adjacent filaments. Scale bar = 100 μm. (23) Radial-vertical section of the thick crust with well-developed epithallus lacking hair bases or trichocytes. Scale bar = 100 μm.

**Holotype:** *GH0015334*, 5.0 m depth, coll. T. Schils & M. Deinhart, 07.viii.2018 (University of Guam Herbarium; GUAM).

**Type locality:** Adjoulan Point at the mouth of Talofofo Bay (13.33806°N, 144.770278°E), Guam, Mariana Islands, western Pacific Ocean.

**Etymology:** Named after the type locality.

**Distribution:** Known from the type locality, Agat, Tanguisson, and Umatac Bay in Guam.

**Specimen examined:** *GH0015334*, Talofofo Bay, Guam, Mariana Islands, western Pacific Ocean, 5.0 m depth, coll. T. Schils & M. Deinhart, 07.viii.2018.

Thalli were burnt orange to burgundy, tightly adherent, and irregularly lumpy due to irregularities in the substrata (Fig 6). Crusts were calcified throughout and typically closely appressed to the substrate but occasionally free at the margins. Crusts were relatively thick, typically reaching 350–600 μm in thickness. Hypothallial cells were parallel and composed of dorsally inflated oval cells that centrally gave rise to assurgent perithallial filaments. Rhizoids were frequently produced, unicellular, and were 75–100 μm long and 10–14 μm wide (Fig 6). Rhizoids were cut off from the ventral portions of hypothallial cells and emerged from the thick (typically 30–35 μm thick) hypobasal cuticle. Cells of the lower perithallus were also oval, but less dorsally inflated than the hypothallial filaments. Cells of the lower perithallus are typically large (18–32 μm high and 16–22 μm wide) and are connected to adjacent cells commonly by pit connections and occasionally by cell fusions (Fig 6). Cells in the mid-perithallus rapidly decrease in size, similar to what was observed in *R*. *bonairensis* [15]. The epithallus is generally well developed, often comprising at least half of the entire perithallus (Fig 6). Upper perithallial cells were commonly connected by secondary pit connections or fused with adjacent cells. Hair bases or trichocytes were absent in the upper perithallus and the upper perithallial filaments were crowded due to occasional branching in the upper perithallus (Fig 6). Reproductive features were not observed.

*Ramicrusta adjoulanensis* shares morphological characteristics with its close relative *R*. *bonairensis*, such as the significant decrease in cell size in the mid-perithallus, the well-developed epithallus with frequent cell fusions and secondary pit connections, and the lack of hair bases or trichocytes. *Ramicrusta adjoulanensis* was distinguished from *R*. *bonairensis* primarily by its attachment, with crusts that were occasionally free at the margins and by its relatively long rhizoids (75–100 μm long) that were frequently produced and penetrated the thick hypobasal cuticle. These features combined with DNA sequence divergences differentiated *Ramicrusta adjoulanensis* from *R*. *bonairensis* and other species of the genus.

#### *Ramicrusta asanitensis* M.Mills et Schils sp. nov.

[urn:lsid:marinespecies.org:taxname:XXXXXXX]

Fig 7.

**Fig 7:**
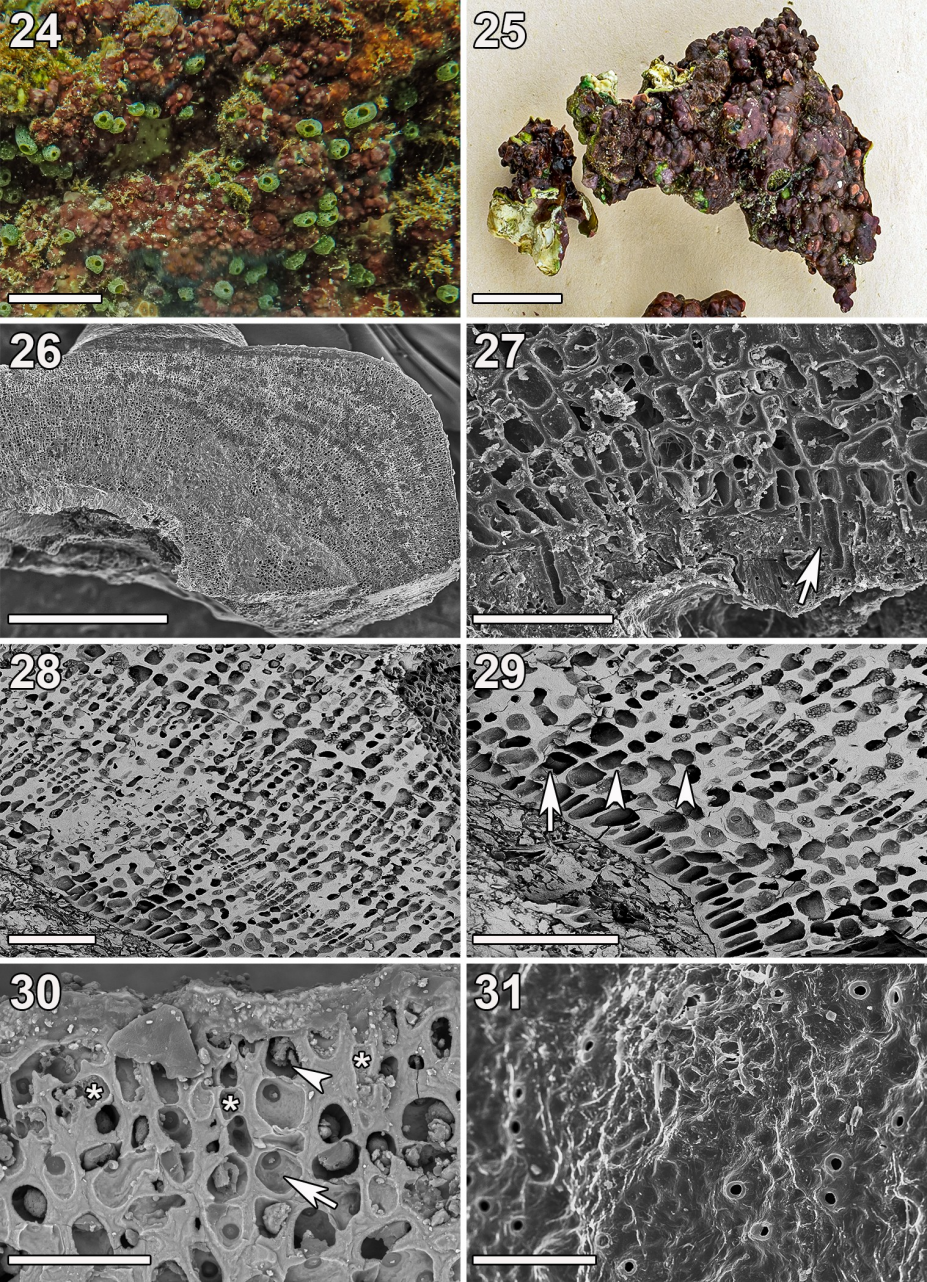
***Ramicrusta asanitensis* (Fig 7: GH0015151; Fig 7: GH0015152).** (24) *In-situ* image of the holotype specimen. Scale bar = 2 cm. (25) Habit of the holotype specimen. Scale bar = 2 cm. (26) Radial-vertical section showing thick crust with multiple layers of growth. Scale bar = 2 mm. (27) Unicellular rhizoids (arrow) penetrating the thin hypobasal cuticle. Scale bar = 50 μm. (28) Radial section showing portions of primary, secondary, and tertiary growth. Scale bar = 100 μm. (29) Radial section showing cell fusions (arrow) and secondary pit connections (arrowheads) in the lower perithallus. Scale bar = 100 μm. (30) Section showing heavily calcified, bullet-shaped hair base or trichocyte (arrowhead) terminating a filament of four cells (arrow) that are significantly larger than other surrounding apical cells (asterisks). Scale bar = 50 μm. (31) Thallus with small, rounded outgrowths on the surface and evidence of multiple hair bases or trichocytes penetrating the epithallus, resulting in small surface pores throughout the thallus surface. Scale bar = 50 μm.

**Holotype:** *GH0015151*, 1.0 m depth, coll. T. Schils & M. Mills, 27.x.2017 (University of Guam Herbarium; GUAM).

**Type locality:** Asanite Cove / First Beach (13.34251°N, 144.77194°E), Guam, Mariana Islands, western Pacific Ocean.

**Etymology:** Named after the type locality, Asanite Cove.

**Distribution:** Known from the type locality and from Pago Bay, Hagåtña Bay and Ipan Beach, Guam.

**Specimens examined:** *GH0015151*, Asanite Cove / First Beach reef flat, Guam, Mariana Islands, western Pacific Ocean, 1.0 m depth, coll. T. Schils & M. Mills, 27.x.2017; *GH0015152*, Asanite Cove / First Beach reef flat, Guam, Mariana Islands, western Pacific Ocean, 1.0–1.5 m depth, coll. T. Schils & M. Mills, 27.x.2017; *GH0015259*, Ipan Beach reef flat, Guam, Mariana Islands, western Pacific Ocean, 0.5–1.0 m depth, coll. T. Schils, M. Deinhart & K. Borja, 18.vi.2018.

Thalli were dark maroon, heavily calcified, and formed closely appressed and tightly adherent crusts on various secondary reef structures such as large rocks and dead corals (Fig 7). The thallus surface contained small, rounded outgrowths, and the crusts were significantly thicker (upwards of 2 mm, but typically 500–1000 μm) than most other *Ramicrusta* species (Fig 7). The hypothallial filaments were parallel and composed of elongate, distally inflated rhomboid to rectilinear cells that gave rise to assurgent perithallial filaments centrally or at variable angles (> 45°). Plants were attached by short (50–80 μm) unicellular rhizoids that were cut off at the distal ventral corners of hypothallial cells and penetrated the thin (10–15 μm thick) hypobasal cuticle (Fig 7). Perithallial filaments were simple, and the perithallus was composed of distinct upper and lower zones. Portions of secondary to tertiary growth as well as overgrowth were present (Fig 7). Secondary and tertiary growth appeared as stacked layers of epithalli and lower perithalli, while overgrowth appeared as one fully formed crust growing atop another. Cells in the lower perithallus were large (15–30 μm long and 12–22 μm wide), distally inflated, and rectilinear to ovoid in shape. These cells were thick walled and heavily calcified, and displayed frequent lateral secondary pit connections or cell fusions (Fig 7). The epithallus was relatively thin and consisted of four to five cell tiers that lacked cell fusions and secondary pit connections. The cells were smaller than those in the lower perithallus but were still thick-walled and heavily calcified (Fig 7). Hair bases or trichocytes were large (20–24 μm long and 11–14 μm wide), bullet-shaped, heavily calcified, and terminated four to five-celled filaments (Fig 7). Hair bases or trichocytes were often, but not always, associated with a pore on the thallus surface (Fig 7). Reproductive features were not observed.

*Ramicrusta asanitensis* possessed certain features similar to those commonly found in other *Ramicrusta* species, namely a closely appressed habit and frequent secondary pit connections and cell fusions in the lower perithallus. In addition, it also possessed the thin epithallus shared with its close relatives *R*. *appressa* and *R*. *fujiiana*. However, *Ramicrusta asanitensis* differed from other *Ramicrusta* species by the heavy calcification in the epithallus (as well as elsewhere in the crust), the thickness of the crust (upwards of 2 mm thick), and the extent of its secondary, tertiary, and quaternary perithallial growth. These features, in conjunction with its distinct genetic sequences, distinguished *Ramicrusta asanitensis* from other *Ramicrusta* species.

#### *Ramicrusta labtasiensis* M.Mills et Schils sp. nov.

[urn:lsid:marinespecies.org:taxname:XXXXXXX]

Fig 8

**Fig 8:**
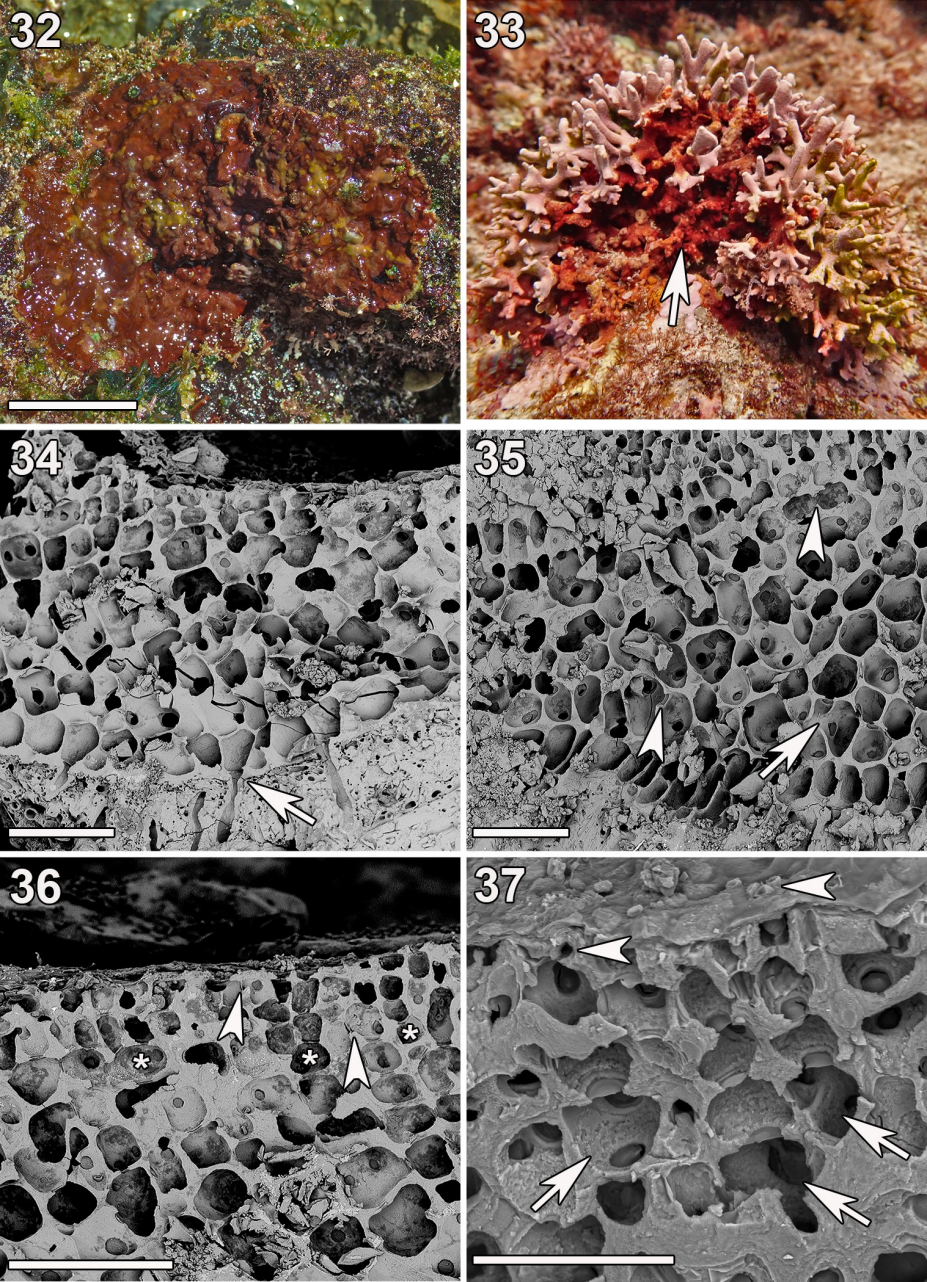
***Ramicrusta labtasiensis* (Fig 8: GH0015097; Fig 8: GH0015717).** (32) *In-situ* image of the holotype specimen displaying one distinct coloration. Scale bar = 2 cm. (33) *In-situ* image of a specimen displaying the other observed coloration (arrow). (34) Crust attached by frequent unicellular rhizoids (arrow). Scale bar = 100 μm. (35) Radial-vertical section showing the irregularly-branching lower perithallus with frequent secondary pit connections (arrowheads) and cell fusions (arrow). Scale bar = 100 μm. (36) Radial section showing the thin epithallus with occasional secondary pit connections (arrowheads) and pairs of filaments borne from the same cell (asterisks). Scale bar = 100 μm. (37) Oblique view of the perithallus showing bullet-shaped hair bases (arrows) and associated surface pores (arrowheads). Scale bar = 50 μm.

**Holotype:** *GH0015097*, 0.5 m depth, coll. T. Schils & M. Mills, 28.ix.2017 (University of Guam Herbarium; GUAM).

**Type locality:** Pago Bay (13.42738°N, 144.798922°E), Guam, Mariana Islands, western Pacific Ocean.

**Etymology:** Named after the type locality, the seawater intake channel behind the University of Guam Marine Laboratory, Pago Bay, Guam. The CHamoru name for Marine Lab was chosen to celebrate the 50^th^ anniversary of the laboratory and to honor the continued support that the institute has received from the island community, the Government of Guam, and the University of Guam.

**Distribution:** Known from the type locality, Lafac Bay, Tumon Bay, and Ritidian Point in Guam.

**Specimens examined:** *GH0015097*, Pago Bay reef flat behind the Marine Laboratory, Guam, Mariana Islands, western Pacific Ocean, 0.5 m depth, coll. T. Schils & M. Mills, 28.ix.2017; *GH0015717*, Lafac Bay, Guam, Mariana Islands, western Pacific Ocean, 6.6 m depth, coll. T. Schils & M. Mills, 31.ix.2019.

Observed thalli expressed two distinct colorations. Thalli were either reddish brown to maroon, with patches of lighter brown scattered throughout, or burnt orange to maroon (Fig 8). Plants were brittle, closely appressed and tightly adherent to dead coral substrate or other calcifying red algae, and formed crusts that were 240–500 μm thick. Hypothallial filaments were parallel and composed of dorsally inflated cells that gave rise to assurgent perithallial filaments centrally or at broad angles. Plants were frequently attached by unicellular rhizoids (75–95 μm long and 10–14 μm wide) that cut off the distal ventral portion of hypothallial cells and penetrated the thin (12–15 μm) hypobasal cuticle (Fig 8). Cells of the lower perithallus were rounded, generally slightly elongate, and formed perithallial filaments that were often irregularly branched. Cells were heavily calcified and were frequently connected to adjacent cells via secondary pit connections or cell fusions (Fig 8). The epithallus was thin and was composed of two to four tiers of small rectilinear cells that were occasionally connected to cells of adjacent filaments via cell fusions or secondary pit connections. Pairs of upper perithallial filaments were often borne from the same cell in the mid-perithallus, resulting in filament crowding in the epithallus (Fig 8). Hair bases were infrequent, but often observed in close proximity to one another. Hair bases were bullet shaped, 23–27 μm long and 14–19 μm wide, and terminated filaments of three to four cells that were typically, but not always, associated with a pore on the thallus surface (Fig 8). Reproductive features were not observed.

*Ramicrusta labtasiensis* shared features with its close relative *R*. *lateralis* such as frequent cell fusions and irregular branching of filaments in the lower perithallus. *Ramicrusta labtasiensis* was primarily distinguished from *R*. *lateralis* by its attachment, where the thallus was frequently attached by robust, relatively long rhizoids (75–95 μm long) throughout its entire undersurface. It is also distinguished by the frequent branching and occasional secondary pit connections and cell fusions in the relatively thin epithallus. These features in combination with the distinct DNA sequences differentiate *Ramicrusta labtasiensis* from other *Ramicrusta* species.

#### *Ramicrusta taogamensis* M.Mills et Schils sp. nov.

[urn:lsid:marinespecies.org:taxname:XXXXXXX]

Fig 9.

**Fig 9:**
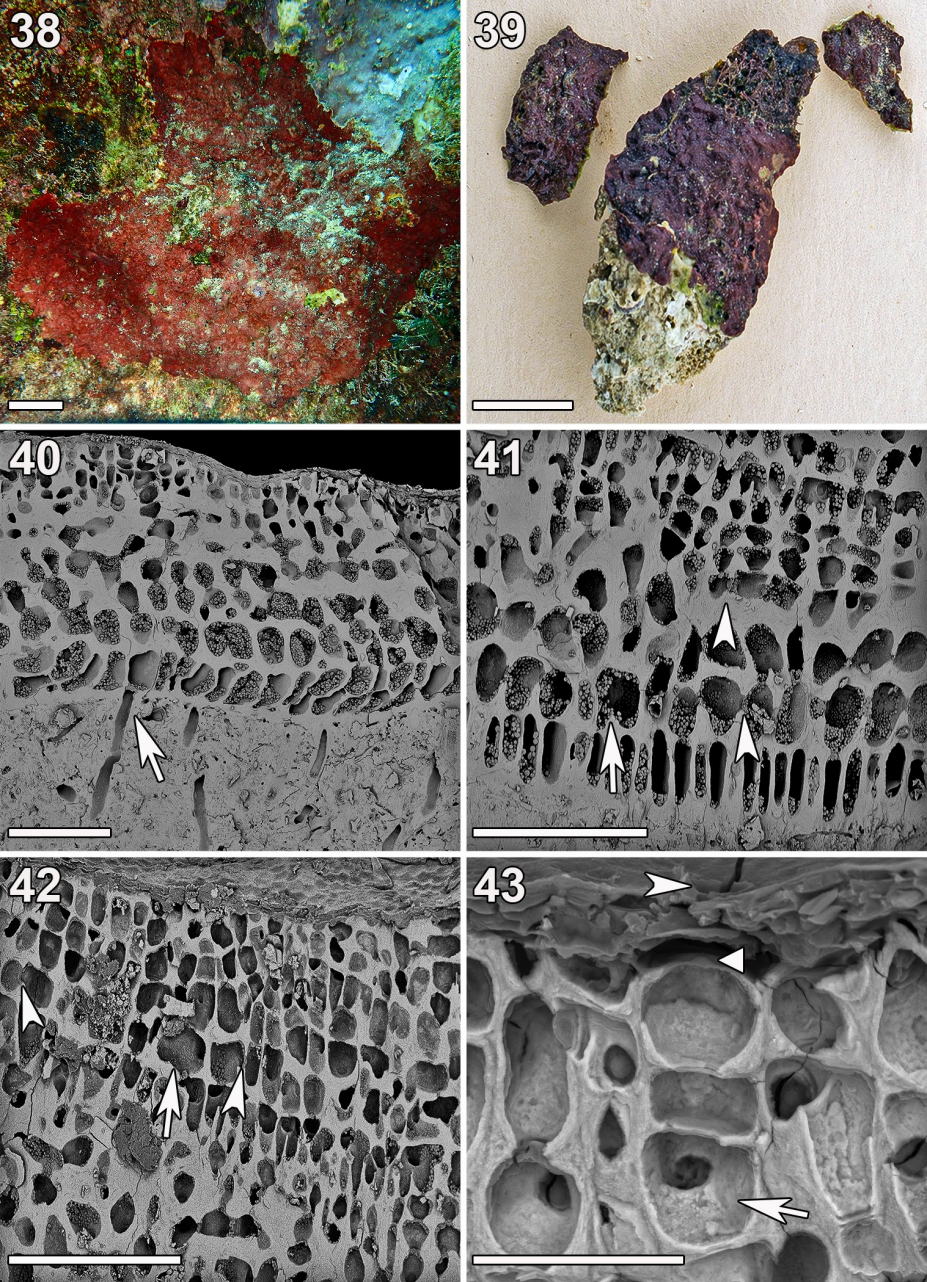
*Ramicrusta taogamensis* (GH0015094). (38) *In-situ* image of the holotype specimen. Scale bar = 2 cm. (39) Habit of the holotype specimen. Scale bar = 2 cm. (40) Short unicellular rhizoid (arrow) cutting off the distal ventral portion of a hypothallial cell. Scale bar = 100 μm. (41) Radial section showing lower perithallus with frequent cell fusions (arrow) and secondary pit connections (arrowheads). Scale bar = 100 μm. (42) Radial section showing cell fusions (arrow) and secondary pit connections (arrowheads) in the well-developed epithallus. Scale bar = 100 μm. (43) Oblique view of a radial section showing a hair base terminating a three cell filament (arrow), its associated surface pore (arrowhead), and a small projection from which a hair may have once emerged (triangle). Scale bar = 30 μm.

**Holotype:** *GH0015094*, 6.3 m depth, coll. T. Schils & M. Mills, 22.ix.2017 (University of Guam Herbarium; GUAM).

**Type locality:** Pago Bay (13.42664°N, 144.799092°E), Guam, Mariana Islands, western Pacific Ocean.

**Etymology:** Named after the type locality near Taogam Point, Pago Bay, behind the University of Guam Marine Laboratory.

**Distribution:** Known only from the type locality and Bile Bay in Guam.

**Specimens examined:** *GH0015094*, Pago Bay submarine terrace, Guam, Mariana Islands, western Pacific Ocean, 6.3 m depth, coll. T. Schils & M. Mills, 22.ix.2017; *GH0015103*, Pago Bay submarine terrace, Guam, Mariana Islands, western Pacific Ocean, 5.4 m depth, coll. T. Schils & M. Mills, 22.ix.2017.

Thalli were deep red to crimson, heavily calcified, and formed tightly adherent and closely appressed crusts (250–500 μm thick) on bedrock (Fig 9). The thallus surface mimicked that of the substratum. The hypothallial filaments were parallel and composed of dorsally inflated, elongate rhomboid and rectilinear cells that gave rise to assurgent perithallial filaments centrally or at broad angles. Plants were attached by short (c. 70 μm long and c. 16 μm wide) unicellular rhizoids that terminated at the distal ventral portion of hypothallial cells and penetrated the relatively thick (~20 μm) hypobasal cuticle (Fig 9). The perithallus was composed of distinct upper and lower zones, divided by a horizontal linear series of cells that were irregularly shaped and frequently fused with the cells of neighboring filaments. The lower perithallus was largely composed of slightly dorsally elongate, thick-walled, and heavily calcified ovoid cells with frequent cell fusions and secondary pit connections (Fig 9). The upper perithallus (epithallus) was generally well-developed, comprising up to half of the entire perithallus. The upper perithallial cells were smaller and also slightly dorsally ovoid, forming a dorsal cortical layer of typically four to six cells thick. The upper perithallial cells were occasionally connected to adjacent cells by secondary pit connections or cell fusions (Fig 9). Hair bases were 17–22 μm long and 13–16 μm wide, circular to bullet-shaped, and terminated mostly three- to four-celled hair filaments (Fig 9). Reproduction was not observed.

The molecular results suggested that *R*. *taogamensis* was a cryptic sister-species of *R*. *appressa*. *Ramicrusta taogamensis* had much in common with its close relative *R*. *appressa*, such as its frequent cell fusions in the lower perithallus and tight adherence by short rhizoids, but was primarily distinguished by its generally well-developed epithallus with occasional cell fusions and secondary pit connections. The epithallus with distinct horizontal linear series of cells and the thicker hypobasal cuticle are vegetative features that are not collectively shared by any other *Ramicrusta* species. The differences in vegetative anatomy in combination with phylogenetic data distinguish *Ramicrusta taogamensis* from *R*. *appressa* and other *Ramicrusta* species.

## Discussion and conclusions

Dixon and Saunders [13] used 4% K2P COI-5P interspecific variation as a threshold to distinguish *Ramicrusta* species. Most *Ramicrusta* species have been reported to exhibit low (< 1.0%) intraspecific sequence divergence values [13, 15, 19], with *Ramicrusta appressa* K.R.Dixon being the sole exception [13]. There was an average of 11.97% COI-5P sequence divergence between *Ramicrusta* species, ranging from 10.24% (*R*. *appressa*) to 13.99% (*R*. *australica*) divergence. For these analyses, *Ramicrusta lehuensis* was considered as a sister species to *R*. *fujiiana* in recognition of the diagnostic morphological features used to describe *R*. *lehuensis* despite the high COI-5P similarity between both species (< 2% divergence). An in-depth examination of the relationship between both species warrants further study. COI-5P sequences of *R*. *textilis* from Jamaica, Vanuatu, and Taiwan are nearly identical, while *R*. *lateralis* specimens from Guam and Vanuatu only demonstrate an average intraspecific divergence of 0.08% and *R*. *fujiiana* specimens from Guam and Brazil exhibit an average intraspecific divergence of 0.18%. The high sequence similarity within species from distant geographical regions further supports the recognition of four new *Ramicrusta* species from Guam. Specimens of *R*. *appressa* from Australia and the Philippines were 2.04% divergent from the holotype specimen from Vanuatu, leading Dixon and Saunders [13] to conclude that they may represent cryptic sister species. The description of *Ramicrusta taogamensis* renders *R*. *appressa* paraphyletic and thus provides support to recognize the *R*. *appressa* samples from Australia and the Philippines as a distinct, monophyletic species. Average sequence divergence between *Ramicrusta taogamensis* and the holotype specimen of *R*. *appressa* was 2.42%, supporting its taxonomic recognition as a new species. Apart from *R*. *taogamensis*, each new species was separated from its nearest-neighbor by more than 4.9% COI-5P barcode divergence. Low mean intraspecific barcode divergence (0.18% in *R*. *fujiiana*, 0.08% in *R*. *lateralis*, 0.33% in *R*. *adjoulanensis*, 0.00% in *R*. *asanitensis*, 0.14% in *R*. *labtasiensis*, and 0.11% in *R*. *taogamensis*) was also demonstrated for each species with more than one specimen sequences. The new *Ramicrusta* species exhibited 12.34% (*R*. *adjoulanensis*), 12.12% (*R*. *asanitensis*), 12.87% (*R*. *labtasiensis*), and 10.78% (*R*. *taogamensis*) divergence when compared to other sequenced representatives of the genus (Table 1). COI-5P sequences of *R*. *textilis* from Jamaica, Vanuatu, and Taiwan are nearly identical, while *R*. *lateralis* specimens from Guam and Vanuatu only demonstrate an average of 0.16% intraspecific divergence and *R*. *fujiiana* specimens from Guam and Brazil exhibit 0.36% average intraspecific divergence. Such high sequence similarity within species from distant geographical regions further supports the recognition of four new *Ramicrusta* species from Guam.

**Table 1:** Table showing the minimum interspecific divergence and maximum intraspecific divergence (Kimura 2-parameter) of the COI-5P marker for all six *Ramicrusta* species from Guam.

		Kimura 2-Parameter Subst. Model		
Species	N	Min. interspecific divergence (%)	Max. intraspecific divergence (%)	Nearest Neighbor
*Ramicrusta lateralis*	4	8.25	0.16	*Ramicrusta labtasiensis*
*Ramicrusta fujiiana*	4	1.48	0.36	*Ramicrusta lehuensis*
*Ramicrusta asanitensis*	6	8.48	0.00	*Ramicrusta lehuensis*
*Ramicrusta labtasiensis*	7	8.25	0.48	*Ramicrusta lateralis*
*Ramicrusta adjoulanensis*	4	4.97	0.76	*Ramicrusta bonairensis*
*Ramicrusta taogamensis*	3	2.33	0.16	*Ramicrusta appressa*

The number of sequenced specimens per species (N) and the nearest neighbor of each species are also shown.

**Fig 10:**
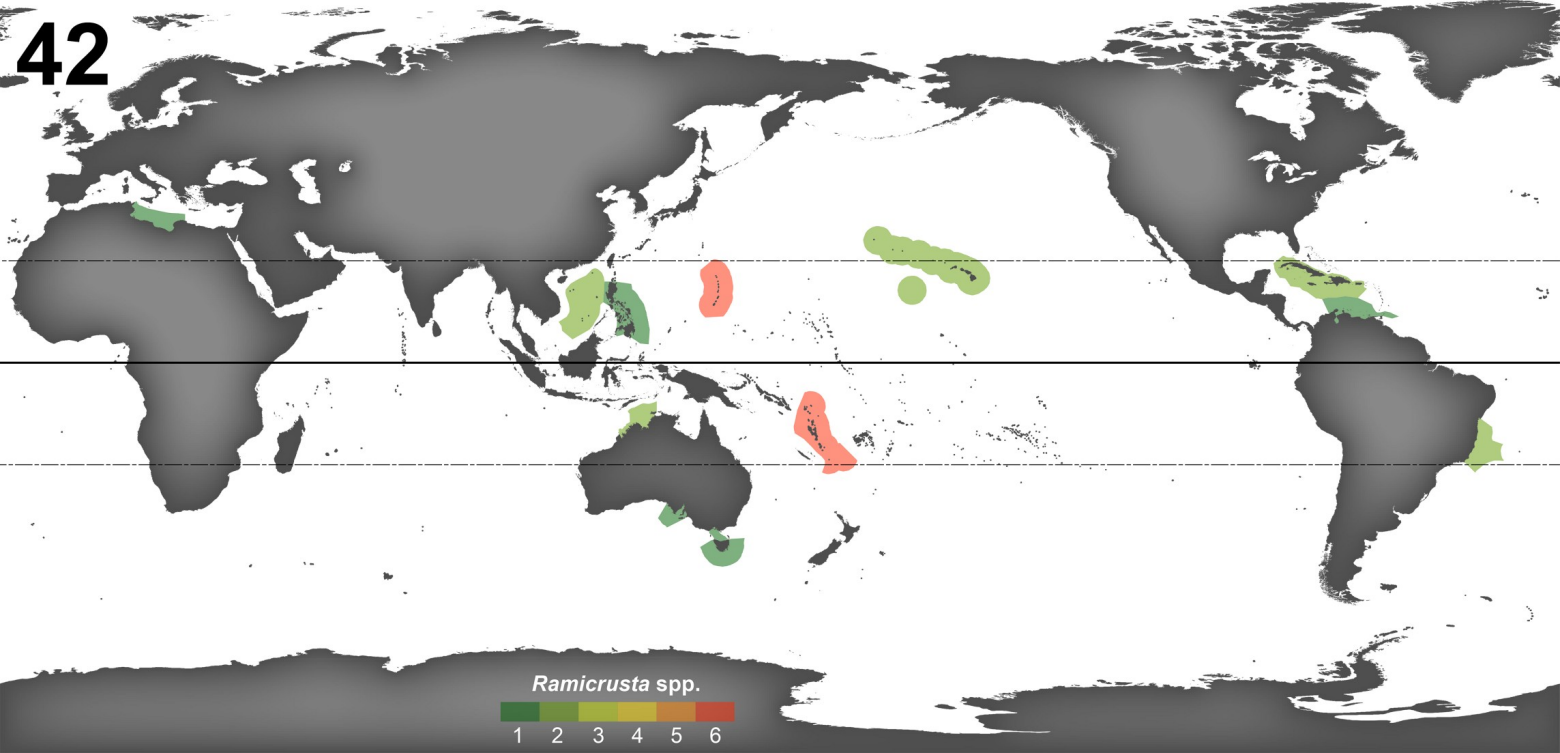
A map of reported *Ramicrusta* species richness by marine ecoregion. The map, created using the ArcGIS computer software, includes all reported *Ramicrusta* species with available DNA sequence data. As with the molecular analyses, *Ramicrusta calcea* was excluded from the map due to its uncertain distribution range and lack of available sequence data.

Crustose calcifying red algae (CCRA) have historically been difficult to identify, largely due to the cryptic diversity and morphological convergence among species [39, 40], as well as their tendency to demonstrate phenotypic plasticity influenced by different environmental factors [41]. As such, studies of CCRA systematics have benefitted greatly from combining molecular methods and anatomical observations [3, 13, 15, 27, 38, 41, 42]. Twenty-four collections from Guam matched the anatomy and morphology of the peyssonnelioid red alga *Ramicrusta*. Anatomical observations paired with DNA sequence analysis revealed the presence of six *Ramicrusta* species. Two of these species corresponded to the previously described species, *R*. *lateralis* and *R*. *fujiiana*, the latter being confirmed by only DNA sequence analysis. Despite its relative abundance on many reefs on Guam, *Ramicrusta* was not known from Guam or anywhere else in the Tropical Northwestern Pacific marine province [27] until now. The COI-5P barcode is widely used to delimit species by employing the barcode gap, and it has been crucial in resolving species boundaries within *Ramicrusta* [13, 15, 19, 22]. *Ramicrusta adjoulanensis*, *R*. *asanitensis*, *R*. *labtasiensis*, and *R*. *taogamensis* exhibited sufficient levels of interspecific divergence to be considered as new species within the genus *Ramicrusta*. The high relative abundance of *Ramicrusta* on certain reefs in Guam may be explained by an increase in disturbance events and an overall decline in reef health over the last decades [43]. Many of the *Ramicrusta* species in Guam were found on reef flats that experience severe fluctuations in temperature, salinity, and nutrients whereas others occurred abundantly on reefs that have been impacted by coral bleaching events or are chronically exposed to pulses of terrestrial runoff. *Ramicrusta* taxa have previously been reported to thrive in disturbed or environmentally stressed reef habitats [14, 15, 26]. There have not been many studies of crustose algae around Guam, and the diversity and ecology of Guam’s CCRA communities are still poorly understood. *Ramicrusta* species have been reported in tropical to temperate waters across the globe. However, because of this study, which was based on a modest sampling effort, Guam now joins Vanuatu in having the highest reported *Ramicrusta* species richness of all marine ecoregions in the world (Fig 10). The recently documented high species richness of these small island nations suggests that the occurrence of *Ramicrusta* species is likely to be severely under-reported globally. The potentially significant ecological impacts of *Ramicrusta* outbreaks on reef health [14, 19, 25, 26] emphasizes the need for further investigations on *Ramicrusta* and CCRA diversity and ecology on a global scale.

## Supporting information

S1 Fig Bayesian inference phylogenetic tree of members of *Ramicrusta* using the *psb*A marker with bootstrap proportions and Bayesian support values. Specimens being described are in bold type.
(TIF)

S2 Fig Bayesian inference phylogenetic tree of members of *Ramicrusta* using the *rbc*L marker with bootstrap proportions and Bayesian support values. Specimens being described are in bold type.
(TIF)

S1 Table Species and sources of sequences used in the phylogenetic analyses. (DOCX)
