# Supplementary figures and images for: The habitat-modifying red alga Ramicrusta on Pacific reefs: A new generic record for the Tropical Northwestern Pacific and the description of four new species from Guam
Source: PLoS One. 2021 Nov 15;16(11):e0259336. doi: 10.1371/journal.pone.0259336 (PMC8592442; doi:10.1371/journal.pone.0259336)

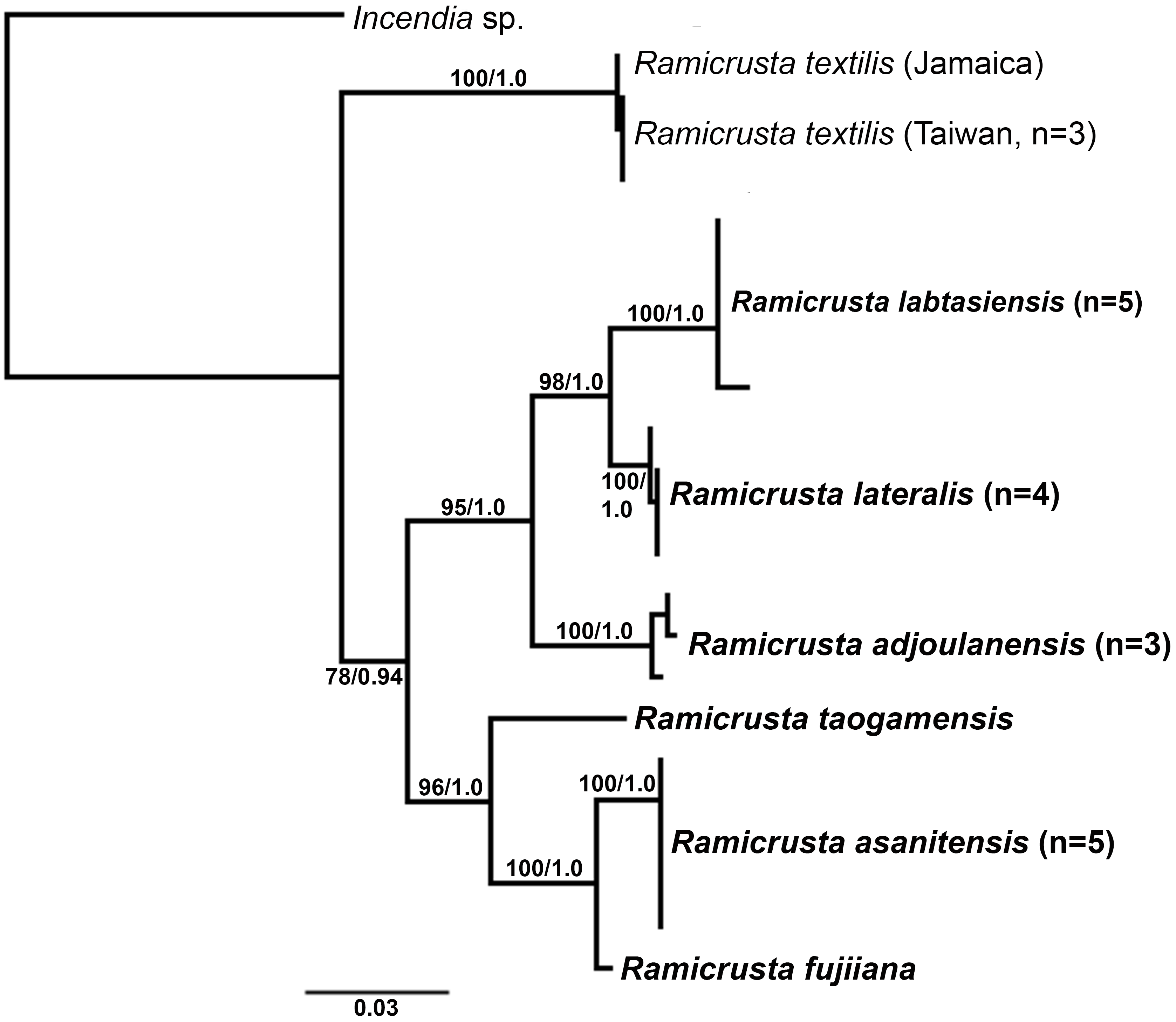

Supplement: S1 Fig — Specimens being described are in bold type. (TIF) [file pone.0259336.s001.tif]

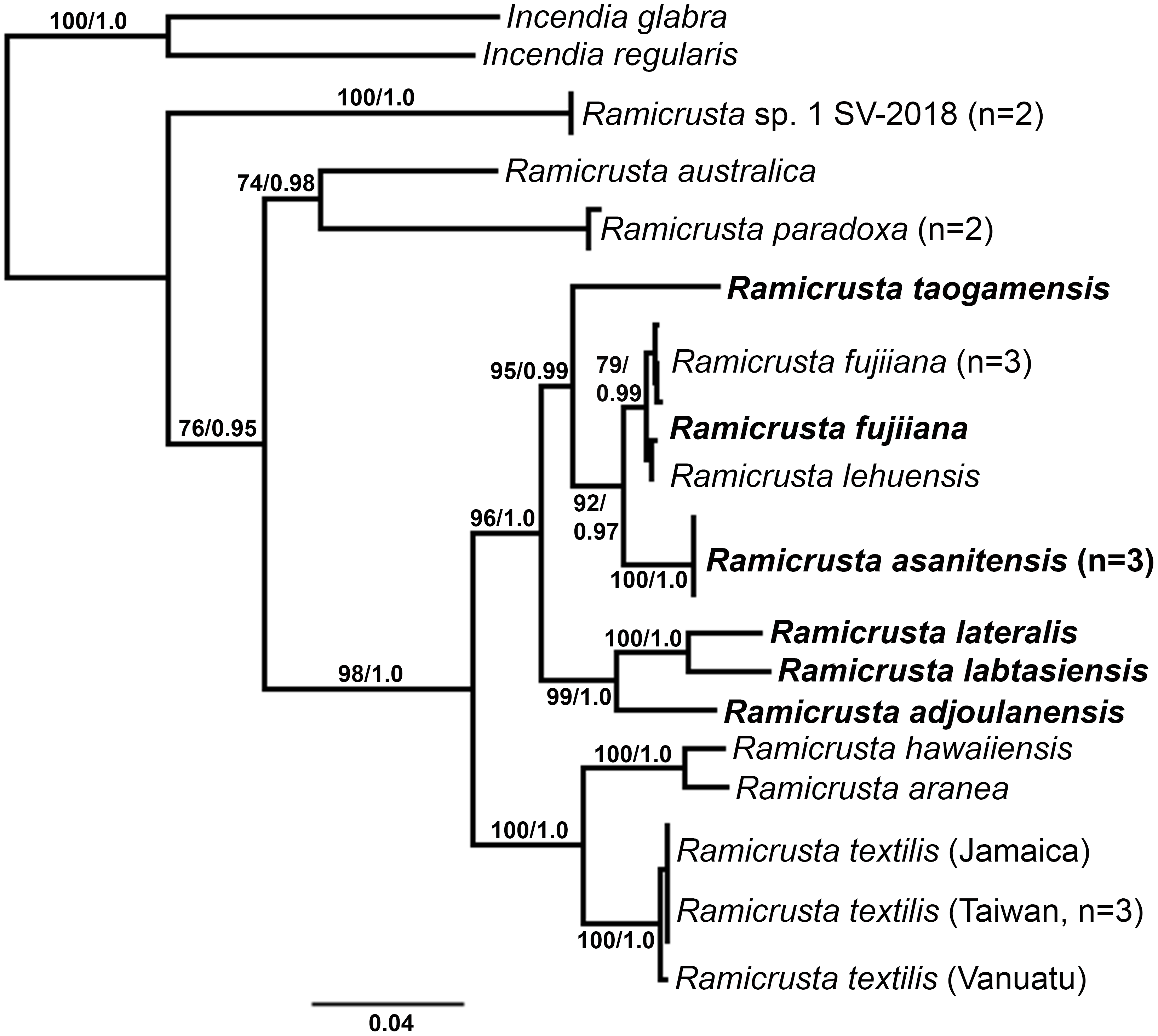

Supplement: S2 Fig — Specimens being described are in bold type. (TIF) [file pone.0259336.s002.tif]
