# Supplementary material for: The habitat-modifying red alga Ramicrusta on Pacific reefs: A new generic record for the Tropical Northwestern Pacific and the description of four new species from Guam
Source: PLoS One. 2021 Nov 15;16(11):e0259336. doi: 10.1371/journal.pone.0259336 (PMC8592442; doi:10.1371/journal.pone.0259336)
Supplement: S1 Table — (DOCX) [file pone.0259336.s003.docx]

|  | GenBank accession number | | |  |
| --- | --- | --- | --- | --- |
| Organism | COI-5P | *rbc*L | *psb*A | Reference |
| *Incendia glabra* | JX969688 | JX969774 |  | [14] |
| *Incendia regularis* | JX969750 | JX969803 |  | [14] |
| *Incendia* sp. 1Cocos |  |  | XX | Unpublished data |
| ***Ramicrusta adjoulanensis* (GH0015334)** | **MW960726** | **MW960752** | **MW960765** | **This study** |
| ***Ramicrusta adjoulanensis* (GH0015394)** | **MW960727** |  |  | **This study** |
| ***Ramicrusta adjoulanensis* (GH0015525)** | **MW960728** |  | **MW960766** | **This study** |
| ***Ramicrusta adjoulanensis* (GH0015652)** | **MW960729** |  | **MW960767** | **This study** |
| *Ramicrusta appressa* | HM918340 |  |  | [14] |
| *Ramicrusta appressa* | JX969695 |  |  | [14] |
| *Ramicrusta appressa* | JX969707 |  |  | [14] |
| ***Ramicrusta asanitensis* (GH0015054)** | **MW960730** | **MW960754** | **MW960761** | **This study** |
| ***Ramicrusta asanitensis* (GH0015060)** | **MW960731** | **MW960755** |  | **This study** |
| ***Ramicrusta asanitensis* (GH0015151)** | **MW960732** |  | **MW960764** | **This study** |
| ***Ramicrusta asanitensis* (GH0015152)** | **MW960733** | **MW960756** | **MW960763** | **This study** |
| ***Ramicrusta asanitensis* (GH0015259)** | **MW960734** |  | **MW960760** | **This study** |
| ***Ramicrusta asanitensis* (GH0015291)** | **MW960735** |  | **MW960762** | **This study** |
| *Ramicrusta aranea* | JX969701 | JX969780 |  | [14] |
| *Ramicrusta australica* | JX969724 | JX969787 |  | [14] |
| *Ramicrusta bonairensis* | KX417374 |  |  | [19] |
| *Ramicrusta fujiiana* | MN990086 | MN990099 |  | [22] |
| *Ramicrusta fujiiana* | MN990087 | MN990100 |  | [22] |
| *Ramicrusta fujiiana* | MN990088 | MN990102 |  | [22] |
| ***Ramicrusta fujiiana* (GH0015078)** | **MW960736** | **MW960757** | **MW960759** | **This study** |
| *Ramicrusta hawaiiensis* | MN623629 | MN623630 |  | [23] |
| ***Ramicrusta labtasiensis* (GH0015097)** | **MW960737** | **MW960751** | **MW960776** | **This study** |
| ***Ramicrusta labtasiensis* (GH0015399)** | **MW960738** |  |  | **This study** |
| ***Ramicrusta labtasiensis* (GH0015524)** | **MW960739** |  | **MW960773** | **This study** |
| ***Ramicrusta labtasiensis* (GH0015617)** | **MW960740** |  | **MW960774** | **This study** |
| ***Ramicrusta labtasiensis* (GH0015717)** | **MW960741** |  |  | **This study** |
| ***Ramicrusta labtasiensis* (GH0015719)** | **MW960742** |  | **MW960775** | **This study** |
| ***Ramicrusta labtasiensis* (GH0015723)** | **MW960743** |  | **MW960772** | **This study** |
| *Ramicrusta lateralis* | JX969721 |  |  | [14] |
| ***Ramicrusta lateralis* (GH0015072)** | **MW960744** | **MW960750** | **MW960770** | **This study** |
| ***Ramicrusta lateralis* (GH0015212)** |  |  | **MW960769** | **This study** |
| ***Ramicrusta lateralis* (GH0015230)** | **MW960745** |  | **MW960768** | **This study** |
| ***Ramicrusta lateralis* (GH0015631)** | **MW960746** |  | **MW960771** | **This study** |
| *Ramicrusta lehuensis* | MN623631 | MN623632 |  | [23] |
| *Ramicrusta monensis* | KX417375 |  |  | [19] |
| *Ramicrusta nanhaiensis* | JX969713 |  |  | [15] |
| *Ramicrusta paradoxa* | MN990091 | MN990103 |  | [22] |
| *Ramicrusta paradoxa* | MN990092 | MN990104 |  | [22] |
| *Ramicrusta* sp. 1 SV-2018 |  | LT969692 |  | Unpublished data |
| *Ramicrusta* sp. 1 SV-2018 |  | LT969712 |  | Unpublished data |
| *Ramicrusta* sp. |  | MG030799 |  | [24] |
| ***Ramicrusta taogamensis* (GH0015094)** | **MW960747** | **MW960753** | **MW960758** | **This study** |
| ***Ramicrusta taogamensis* (GH0015103)** | **MW960748** |  |  | **This study** |
| ***Ramicrusta taogamensis* (GH0015641)** | **MW960749** |  |  | **This study** |
| *Ramicrusta textilis* | JX969749 | KC130226 | KM360015 | [16] |
| *Ramicrusta textilis* | JX969704 |  |  | [14] |
| *Ramicrusta textilis* | JX969690 | JX969775 |  | [14] |
| *Ramicrusta textilis* | KX417373 |  |  | [19] |
| *Ramicrusta textilis* | MK616539 | MK616531 | MK616535 | [18] |
| *Ramicrusta textilis* | MK616540 | MK616532 | MK616536 | [18] |
| *Ramicrusta textilis* | MK616538 | MK616530 | MK616534 | [18] |
| *Ramicrusta trichaurea* | JX969719 |  |  | [14] |
